# Bacterial community shifts in *Fusarium*-induced avocado root rot and the antagonistic potential of *Bacillus siamensis* NB92

**DOI:** 10.3389/fmicb.2025.1626537

**Published:** 2025-06-18

**Authors:** Chengxian Wang, Zhijiao Song, Xue Li, Qing Liu

**Affiliations:** ^1^School of Resources and Environment, Baoshan University, Baoshan, China; ^2^Baoshan Key Laboratory of Biodiversity Conservation and Utilization of Gaoligong Mountains, Baoshan, China; ^3^Research Institute of Gaoligong Mountains, Baoshan University, Baoshan, China

**Keywords:** avocado root rot, *Fusarium*, rhizosphere microbiome, bacterial community dynamics, *Bacillus siamensis*, biological control

## Abstract

Avocado root rot, driven by soil-borne fungi such as *Fusarium* spp., poses a major challenge to global avocado production. The rhizosphere microbiome is critical for plant health, yet the impact of root rot on bacterial community structure and its implications for disease management remain poorly understood. Here, we combined culture-independent 16S rDNA amplicon sequencing with culture-dependent isolation to characterize bacterial communities in healthy and *Fusarium*-infested avocado bulk and rhizosphere soils. Key beneficial taxa, notably *Bacillus*, were then isolated and evaluated for their antagonistic potential. Results showed that root rot significantly reduced rhizosphere bacterial α-diversity, altered community structure, and depleted phyla such as Actinobacteriota and Firmicutes that contain beneficial taxa. Beneficial genera such as *Bacillus* and *Streptomyces* declined, while cultivable *Fusarium* counts increased. Negative correlations between *Fusarium* abundance, the bacteria-to-fungi ratio, and the relative abundance of beneficial bacteria further underscore their suppressive role. Guided by these findings, we isolated *Bacillus* strain NB92, identified as *Bacillus siamensis* through morphological, biochemical, and 16S rRNA and *gyrA* gene analyses. NB92 exhibited strong antagonistic activity against the root rot pathogen (*Fusarium* sp. St7) via both direct antagonism and volatile organic compound production. Inoculating NB92 into diseased rhizosphere soil boosted *Bacillus* counts and reduced *Fusarium* abundance. Moreover, NB92 effectively inhibited the pathogen’s necrotizing ability. *B. siamensis* NB92 thus represents a promising, sustainable biocontrol agent and contributes to the development of microbiome-based strategies for managing avocado root rot.

## 1 Introduction

Root rot is a major plant disease that threatens global agricultural production, affecting a wide range of economically valuable crops, including medicinal plants, legumes, and fruit trees (Williamson-Benavides and Dhingra, 2021). Among these, avocado (*Persea americana* Mill.) root rot—primarily caused by soil-borne fungi and oomycetes including *Phytophthora* spp., *Fusarium* spp., and *Cylindrocarpon* spp.—presents a particularly severe threat to global avocado cultivation (Pagliaccia et al., 2013; Aiello et al., 2020; Solís-García et al., 2020). The unique flavor and nutritional profile of avocados have driven growing global demand (Reverchon et al., 2023). However, intensified cultivation, particularly in regions like Menglian County and Longling County in Yunnan Province, China, has led to a surge in root rot cases. This disease not only compromises plant health and fruit quality but also results in significant economic losses. Traditional control methods, such as chemical treatments and conventional agronomic practices, often face environmental drawbacks and limited long-term efficacy (Belisle et al., 2019; Farooq et al., 2022). Therefore, there is an urgent need to develop environmentally friendly and highly effective biocontrol systems that target pathogen suppression and activate soil disease-suppressive functions, reducing chemical dependency while enhancing control efficiency.

In the highly complex soil ecosystem, the dynamic balance among microbial communities is fundamental to plant vitality (Compant et al., 2025). These microbial communities interact through both cooperative and competitive mechanisms that shape ecological niches and regulate nutrient availability (Raaijmakers et al., 2009). The rhizosphere, a dynamic microecosystem formed by plant roots, soil, and their associated microorganisms, is often referred to as the plant’s “second genome” due to its key role in controlling carbon and nitrogen cycling and mediating resistance to soil-borne pathogens (Berendsen et al., 2012). However, root rot profoundly disrupts these microbial communities by increasing the diversity and relative abundance of pathogenic taxa, while simultaneously disturbing the stable, disease-suppressive constituents essential for soil health (Raaijmakers et al., 2009).

Plant roots interact with a wide array of microorganisms, including bacteria, fungi, and oomycetes, where fungi and oomycetes are often the primary contributors to severe diseases. Bacterial communities typically show a negative correlation with eukaryotic microbes, serving as defenders against virulent root-associated eukaryotes (Duran et al., 2018). While certain fungal communities can improve nutrient availability for host plants and contribute to disease resistance (Bonfante and Genre, 2010; Wang et al., 2024a), the relatively stable structure of bacterial communities provides a more reliable foundation for sustainable biocontrol strategies. In contrast, the more variable nature of fungal communities underscores the need for more targeted interventions in fungal disease management (Duran et al., 2018). Beneficial bacteria suppress fungal pathogens through mechanisms such as direct antagonism, competition for nutrients and space, enhancing nutrient uptake, and inducing plant defense responses (Zhang et al., 2023). For example, indigenous bacterial populations in the tobacco rhizosphere have effectively controlled fungal wilt (Santhanam et al., 2015), and synthetic communities based on *Bacillus* spp. isolated from tomato roots have significantly reduced *Fusarium* wilt incidence (Tsolakidou et al., 2019). These findings highlight the potential of harnessing beneficial bacteria as a sustainable biocontrol strategy against avocado root rot.

Comparative analyses of bacterial communities in healthy and root rot–affected soils are expected to yield critical insights into how beneficial microbes confer resistance to pathogens, thereby facilitating the development of effective biocontrol strategies. In healthy soils, stable bacterial populations likely serve as reservoirs of disease-suppressive functions, whereas disruptions in these communities in diseased soils may facilitate pathogen proliferation (Fang et al., 2024; Wang et al., 2024b). Despite this, a comprehensive comparison of bacterial communities in healthy versus diseased avocado rhizospheres—and a clear understanding of the specific impacts of root rot on bacterial community dynamics—remains lacking.

Based on these considerations, we hypothesize that avocado root rot disrupts the native bacterial community, resulting in a decline in beneficial taxa essential for maintaining ecological balance and suppressing pathogenic fungi. Specifically, we expect healthy avocado soils to harbor a beneficial and functionally diverse bacterial community that promotes disease resistance, whereas diseased soils will exhibit reduced diversity and an altered community composition that favors pathogen proliferation. To test this hypothesis, our study combines culture-independent high-throughput sequencing with culture-dependent isolation techniques to compare the bacterial community structures of both healthy and *Fusarium*-induced root rot-affected avocado bulk and rhizosphere soils, with particular emphasis on isolating and characterizing key beneficial bacteria, such as *Bacillus*. By elucidating the shifts in bacterial community composition, diversity, and biocontrol potential associated with avocado root rot, our research aims to provide new insights into the microbial mechanisms driving disease progression and offers a foundation for developing targeted, ecologically sustainable biocontrol strategies.

## 2 Materials and methods

### 2.1 Soil sampling

Soil samples were collected from an avocado plantation in Longling County (24°20′N, 99°8′E), Baoshan City, Yunnan Province, China. This subtropical region has an average elevation of approximately 1,600 m, an annual mean temperature of about 17°C, and an average annual precipitation of approximately 1,400 mm. Sampling commenced when the avocado plants were 4 years old, coinciding with the initial appearance of root rot symptoms—including root corrosion, leaf yellowing, wilting, and stunted growth. Soil analysis revealed a *Fusarium* spp. density of 3.4 × 10^6^ CFU/g dry soil, with *Fusarium* sp. st7, isolated from decayed avocado roots, identified as the predominant fungal pathogen. From September 2023 to July 2024, bulk and rhizosphere soil samples were collected from 10 healthy and 10 root rot–diseased avocado plants selected based on visual disease symptoms. The samples were categorized into four groups: rhizosphere soil from diseased plants (BFDR), bulk soil from diseased plants (BFDB), rhizosphere soil from healthy plants (BFHR), and bulk soil from healthy plants (BFHB). To minimize spatial heterogeneity, five biological replicates were created for each group by combining two sub-replicates (individual plants) into one composite sample, yielding a total of 20 samples for subsequent microbial analyses. Soil sampling was performed according to the method described by Bulgarelli et al. (2012).

### 2.2 Quantification of cultivable soil microorganisms

To quantify cultivable microorganisms in soil, 5 g of soil was mixed with 45 mL of sterile water and subjected to tenfold serial dilutions (10^−1^ to 10^−7^). Aliquots from the appropriate dilutions were spread evenly onto different media and incubated under specified conditions. Bacterial colonies were counted on Nutrient Agar (NA) (10.0 g peptone, 3.0 g beef extract, 5.0 g NaCl, and 15.0 g agar per liter water, pH 7.0) after incubation at 30°C for 1–2 days. Fungal colonies were enumerated on Rose Bengal Medium (RBM) (5.0 g peptone, 10.0 g dextrose, 1.0 g monopotassium phosphate, 0.5 g magnesium sulfate, 15.0 g agar, 0.0133 g Rose Bengal, and 0.1 g chloramphenicol per liter water, pH 6.0 ± 0.2) after incubation at 28°C for 2–3 days. *Fusarium* spp. were quantified on Komada selective medium (Komada, 1975) following incubation at 28°C for 5–7 days. Colony-forming units (CFUs) were counted, and the mean values were calculated from five replicate measurements. The results are expressed as CFU per gram of dry soil.

### 2.3 Microbial community sequencing and analysis

Soil samples were processed to extract total metagenomic DNA using the PowerSoil^®^ DNA Isolation Kit (MoBio Laboratories, Carlsbad, CA, United States). DNA concentrations were quantified using a NanoDrop ND-1000 spectrophotometer (Thermo Fisher Scientific, Waltham, MA, United States). PCR amplification was performed targeting the V3–V4 region of the bacterial 16S rRNA gene using primers 341F/806R (Takahashi et al., 2014). The PCR reactions were carried out using Phusion^®^ High-Fidelity PCR Master Mix (New England Biolabs, Ipswich, MA, United States) under the following conditions: initial denaturation at 95°C for 2 min, followed by 30 cycles of denaturation at 95°C for 30 s, annealing at 55°C for 30 s, and extension at 72°C for 1 min, with a final extension at 72°C for 10 min. The PCR products were then purified using the GeneJET™ Gel Extraction Kit (Thermo Fisher Scientific) and sequenced on an Illumina MiSeq platform (San Diego, CA, United States) using paired-end 250 bp reads. Sequencing data have been deposited in the NCBI Sequence Read Archive (accession number: PRJNA1247462).

For bioinformatic analysis, we used USEARCH11 to remove barcodes, primers, and low-quality reads before concatenating the sequences (Edgar, 2018). Zero-radius operational taxonomic units (zOTUs) were generated using the UNOISE3 algorithm, which also removed chimeras and low-abundance sequences (<8 reads). Taxonomic assignments were made by aligning the sequences with the bacterial Silva database (Quast et al., 2013). To account for variation in sequencing depth, zOTU abundances were rarefied to the smallest sample size (29,300 sequences). Microbial *α*-diversity was assessed using Shannon indices, while microbial β-diversity was evaluated through principal coordinate analysis (PCoA) and hierarchical cluster analysis based on Bray-Curtis distance. These analyses were visualized using ggplot2 (v3.5.1) in R software (v4.3.1). Differences in microbial community structure among groups were assessed using PERMANOVA with 999 permutations, implemented via the adonis function in the vegan package (v2.5–7) in R. To distinguish true biological differences from potential dispersion effects, we further conducted a permutational analysis of multivariate dispersions (BETADISP) using the betadisper and permutest functions with 999 permutations. Differentially abundant taxa were then identified using LEfSe (Linear Discriminant Analysis Effect Size), with a significance threshold of LDA > 3.5 (Segata et al., 2011).

### 2.4 Isolation and characterization of antagonistic *Bacillus* strains

A 1 g sample of healthy rhizosphere soil was added to 9 mL of a 0.1% (w/v) sodium pyrophosphate solution and evenly mixed. The soil suspension was heated at 80°C for 15 min in a water bath and then serially diluted from 10^−1^ to 10^−6^, and 0.1 mL aliquots from the 10^−4^ to 10^−6^ dilutions were plated onto NA media. Plates were incubated at 25°C and monitored for bacterial growth over 1–2 days. *Bacillus*-like colonies were selected, transferred to fresh NA plates, and incubated at 30°C for further purification through repeated subculturing.

The antagonistic activity of the *Bacillus* isolates against the root rot pathogen (*Fusarium* sp. St7) was evaluated using a modified dual-culture assay (Andrade-Hoyos et al., 2020). Briefly, a 5 mm mycelial block of *Fusarium* was placed at the center of a 9 cm Petri dish containing Potato Dextrose Agar (PDA). The *Bacillus* isolates were streaked equidistantly (25 mm) on either side of the pathogen plug. Control plates (CK) contained only the *Fusarium* sp. St7 inoculum. Four replicates were prepared per treatment, and plates were incubated at 28°C for 5 days. Mycelial growth was measured by determining the colony diameter, and the growth inhibition rate (%) was calculated as:


$$\frac{\text{Control colony diameter}-\text{Treatment colony diameter}}{\text{Control colony diameter}\;}\times 100$$

The effect of the screened *Bacillus* strain (NB92) fermentative liquid on *Fusarium* sp. St7 conidia germination was tested as described previously (Wang et al., 2023). The conidia germination rate (%) was calculated as:


$$\frac{\text{Number of germinated conidia}}{\text{Number of counted conidia}}\times 100$$

Additionally, the inhibitory effect of NB92-derived VOCs on *Fusarium* sp. St7 was evaluated using a modified two-sealed plate assay (Wang et al., 2012). In the bottom compartment, Nutrient Agar (NA) was overlaid with 100 μL of an overnight NB92 suspension (≈1 × 10^9^ CFU/mL), spread evenly, and air-dried under sterile conditions. The lid compartment received 20 mL of PDA amended with 20 μg/mL chloramphenicol to prevent bacterial carryover. A 5 mm agar plug of actively growing *Fusarium* sp. St7 was placed at the center of the PDA. Dishes were inverted so that the PDA compartment faced the NB92 culture, sealed with Parafilm, and incubated at 28°C for 5 days. Radial fungal growth was then measured and compared to control dishes lacking NB92. This configuration isolates VOC effects—any observed inhibition reflects the cumulative activity of NB92-emitted volatiles without direct bacterial-fungal contact.

The *Bacillus* strain (NB92) was initially characterized through morphological observation and tested for sugar metabolism ability. For molecular identification of the strain, genomic DNA was extracted from the purified isolates, and the 16S rRNA gene was amplified using primers 27F/1492R (Heuer et al., 1997), while the *gyrA* (DNA gyrase subunit A) gene was amplified using primers p-gyrA-f/p-gyrA-r (Chun and Bae, 2000). The resulting amplicons were sequenced by Sanger sequencing (Yunnan Qingshuo Technology Co., Ltd., China) and deposited in GenBank (accession numbers: PV465220-PV465225). Sequence alignments with type strains from the NCBI database were performed using BLASTn, and species were assigned based on the highest similarity. Phylogenetic relationships were further confirmed by constructing a Neighbor-Joining tree with 1,000 bootstrap replicates using MEGA-X software.

### 2.5 Effects of *Bacillus* NB92 application on *Fusarium* suppression

To evaluate the *in vivo* efficacy of *Bacillus siamensis* NB92 in suppressing *Fusarium*, 100 g of rhizosphere soil collected from diseased avocado plants (BFDR) was placed into sterile plastic pots. The soil was then treated with a *B. siamensis* NB92 suspension to achieve a final concentration of 1 × 10^8^ CFU/g dry soil. Pots were maintained under greenhouse conditions (18–27°C) and watered every 2–3 days. In the control group (untreated BFDR), sterile water was applied instead of the bacterial suspension. After 15 days, the number of cultivable *Fusarium* was determined as described in Section 2.2, and the abundance of *Bacillus* was quantified using semi-selective salt-V8 agar medium (Turner and Backman, 1991). Additionally, to assess the inhibitory effect of VOCs emitted from NB92-inoculated soil, a modified two-sealed plate assay was performed as described in Section 2.4. Briefly, 20 g of soil from each treatment group (Control: *Fusarium* only; CK. Soil: soil without NB92; NB92. Soil: NB92-inoculated soil) was evenly spread in the base of a sterile 9 cm Petri dish. The lid was filled with 20 mL of PDA medium supplemented with 20 μg/mL chloramphenicol and inoculated in the center with a 5 mm agar plug of actively growing *Fusarium* sp. St7. The plates were sealed with Parafilm, inverted, and incubated at 28°C for 5 days. Fungal radial growth was measured to evaluate the inhibitory effect of soil-emitted VOCs.

To further assess the biocontrol potential of *Bacillus* NB92 against avocado root rot, an *ex vivo* stem segment inoculation assay was conducted following the method of Solís-García et al. (2020), with slight modifications. Healthy stem segments (~8 cm in length) were excised from cv. *Hass* avocado and surface-sterilized by sequential immersion in 75% ethanol for 1 min, followed by 1% sodium hypochlorite for 3 min. The segments were then rinsed three times in sterile distilled water and air-dried under sterile conditions. The sterilized stem segments were randomly assigned to four treatment groups: (1) CK (control): segments soaked in sterile water for 2 h; (2) NB92: segments soaked in a suspension of NB92 (1 × 10^8^ CFU/mL) for 2 h; (3) St7: segments soaked in sterile water for 2 h, then each cut end was inoculated with a 5 mm mycelial plug of *Fusarium* sp. St7; (4) NB92 + St7: segments soaked in a suspension of NB92 (1 × 10^8^ CFU/mL) for 2 h, then inoculated with *Fusarium* sp. St7 as described above. All treated segments were placed in sterile 9 cm Petri dishes lined with moist filter paper and incubated at 25°C and approximately 80% relative humidity for 15 days. Disease progression was assessed every 3 days by monitoring vascular tissue necrosis, enabling the evaluation of NB92’s protective efficacy against pathogen’s necrotizing ability.

### 2.6 Statistical analysis

Spearman’s correlation analysis was conducted to evaluate relationships among the culturable abundances of bacteria, fungi, and pathogenic *Fusarium*, as well as bacterial community structure (PCo1 scores), α-diversity (Shannon index), and the relative abundances of beneficial bacterial genera. Data normality was assessed using the Shapiro–Wilk test. One-way analysis of variance (ANOVA) followed by Tukey’s Honestly Significant Difference (HSD) test was used to determine significant differences among treatments, with a significance threshold of *p* < 0.05.

## 3 Results

### 3.1 Changes in culturable microorganism abundances

**Figure 1:**
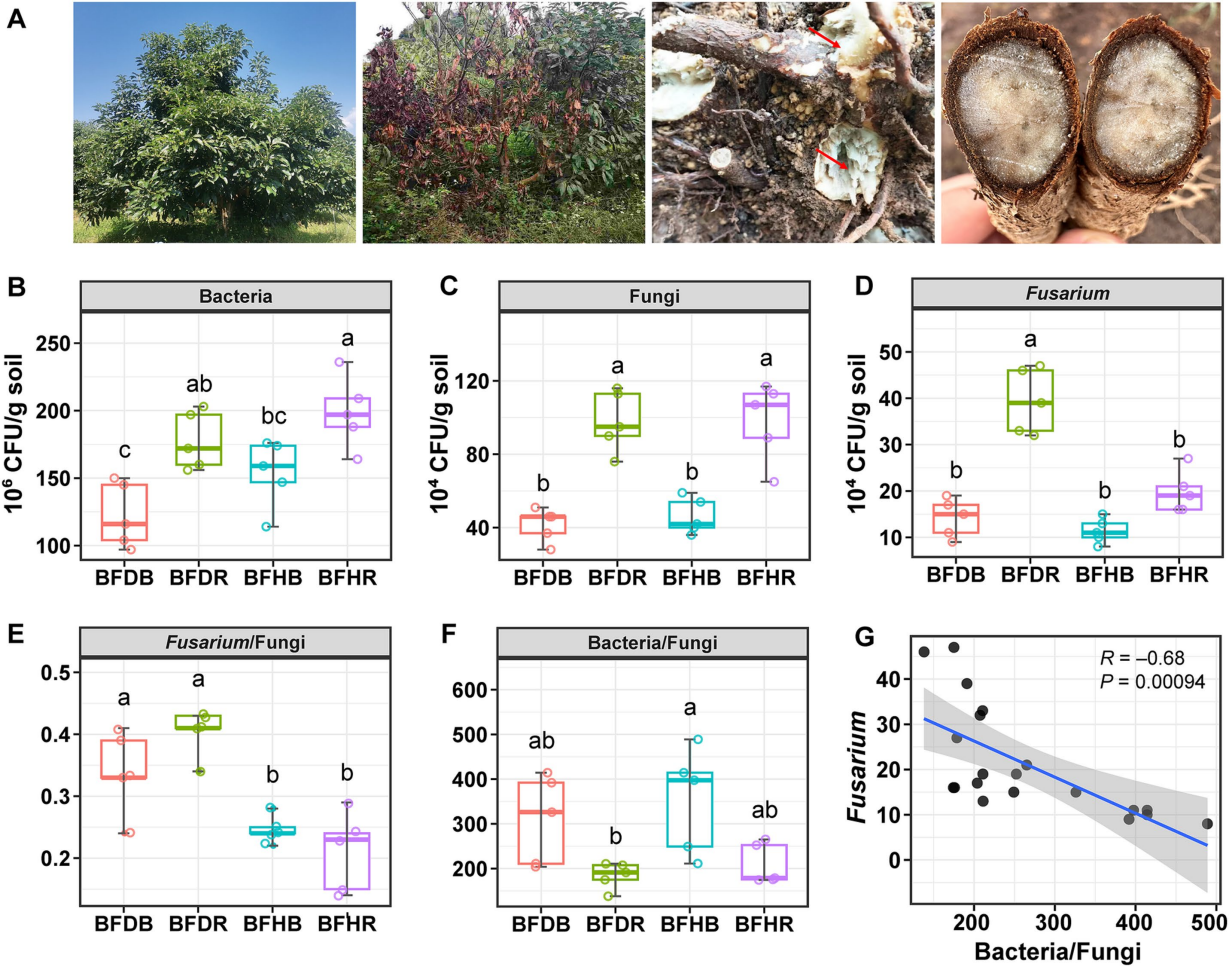
Avocado root rot and the abundance of culturable microorganisms in healthy and diseased soils. **(A)** Typical symptoms of avocado root rot (from left to right): healthy avocado trees; diseased avocado tree showing leaf yellowing, wilting, and stunted growth; diseased avocado tree with rotted roots; diseased avocado tree with rotted stems. **(B)** Number of culturable bacteria, **(C)** fungi, **(D)** *Fusarium*, **(E)** ratio of *Fusarium* to fungi, **(F)** ratio of bacteria to fungi, and **(G)** linear regression analysis between *Fusarium* abundance and the bacteria-to-fungi ratio. The boxplots represent the interquartile range (IQR), with the lower and upper bounds corresponding to the first and third quartiles, respectively. The horizontal line within each box indicates the median. Vertical lines with horizontal caps extending from the boxes represent the minimum and maximum values of the dataset, generated using stat_summary (geom = “errorbar,” fun.min = “min,” fun.max = “max”) in ggplot2, and serve the same function as traditional boxplot whiskers. Different lowercase letters indicate significant differences among treatments at *p* < 0.05, as determined by Tukey’s HSD test. BFDB and BFDR refer to bulk soil and diseased rhizosphere soil, respectively; BFHB and BFHR denote bulk and healthy rhizosphere soil.

Avocado root rot symptoms primarily include root corrosion, leaf yellowing, wilting, and stunted growth of the trees (Figure 1A). In a preliminary experiment, we assessed soil physicochemical properties—such as pH, organic matter content, nitrogen, phosphorus, and potassium levels—and found no significant differences (*p* > 0.05) between healthy and root rot–affected soils (Table 1). Therefore, this study primarily focuses on analyzing microbial community dynamics.

**Table 1:** Soil physicochemical properties.

Parameter	Diseased soil	Healthy soil
pH	5.6 ± 0.16 a	5.72 ± 0.13 a
Total nitrogen (g/kg)	1.4 ± 0.09 a	1.34 ± 0.1 a
Nitrate nitrogen (mg/kg)	79.74 ± 3.11 a	75.17 ± 4.21 a
Ammonium nitrogen (mg/kg)	7.42 ± 0.46 a	6.98 ± 0.68 a
Available potassium (mg/kg)	224.9 ± 19.16 a	216.93 ± 21.3 a
Available phosphorus (mg/kg)	32.54 ± 2.06 a	35.05 ± 1.38 a
Organic matter (g/kg)	19.86 ± 2.53 a	19.25 ± 2.4 a

Values (mean ± SD, *n* = 4) within the same row followed by different lowercase letters indicate significant differences between diseased and healthy soils at *p* < 0.05, as determined by the *t*-test.

The numbers of culturable microorganisms were determined in both bulk and rhizosphere soils collected from healthy and diseased (root rot–affected) avocado plants. Overall, the culturable populations of bacteria, fungi, and *Fusarium* were consistently higher in the rhizosphere than in the corresponding bulk soils (i.e., BFDR > BFDB and BFHR > BFHB; Figures 1B–D). Notably, the highest bacterial counts were recorded in the healthy rhizosphere soil (BFHR), which were significantly greater (*p* < 0.05) than those in both bulk soils (BFDB and BFHB). In contrast, fungal counts did not differ significantly between the healthy and diseased rhizosphere soils (BFHR vs. BFDR). For *Fusarium*, the greatest abundance was observed in the diseased rhizosphere (BFDR), with no significant differences detected among the other groups.

Moreover, the ratio of *Fusarium* to total fungal counts was markedly higher in diseased soils compared to healthy soils, whereas the ratio of bacteria to fungi was elevated in healthy bulk soil (Figures 1E,F). Linear regression analysis further revealed a significant negative correlation between *Fusarium* abundance and the bacteria-to-fungi ratio (*R* = −0.68, *p* = 0.00094; Figure 1G).

### 3.2 Alpha diversity and Venn analysis

High-throughput sequencing of 16S rRNA amplicons was performed to explore the effects of root rot on microbial communities across different soil types (bulk soil, healthy rhizosphere soil, and diseased rhizosphere soil). In total, 842,225 high-quality 16S rRNA sequences were obtained, with each sample yielding between 26,938 and 54,805 sequences. After rarefaction, 3,352 zOTUs were identified, and the rarefaction curves for each sample approached saturation (Supplementary Figure 1), confirming that the sequencing depth was sufficient to capture the microbial diversity and composition. Root rot was found to alter the diversity, structure, and composition of the microbiome.

**Figure 2:**
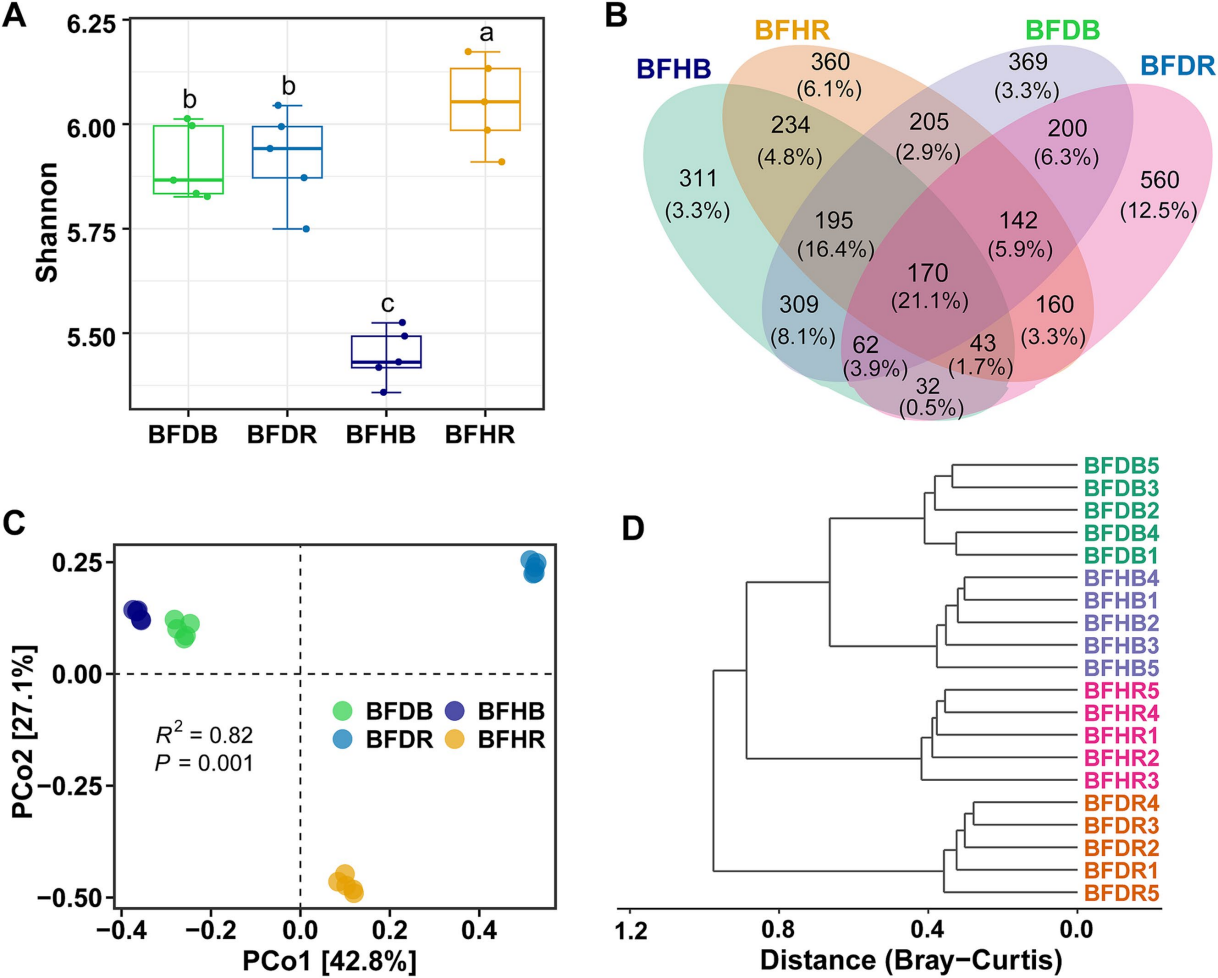
Bacterial community α-diversity and structure across four soil treatments. **(A)** Alpha diversity as measured by the Shannon index. **(B)** Venn diagram showing unique and shared zOTUs among soil compartments. **(C)** Principal coordinate analysis (PCoA) based on Bray–Curtis distances, illustrating microbial community separation. **(D)** Hierarchical clustering of samples using Bray–Curtis dissimilarity. The boxplots represent the interquartile range (IQR), with the lower and upper bounds corresponding to the first and third quartiles, respectively. The horizontal line within each box indicates the median. Vertical lines with horizontal caps extending from the boxes represent the minimum and maximum values of the dataset, generated using stat_summary (geom = “errorbar,” fun.min = “min,” fun.max = “max”) in ggplot2, and serve the same function as traditional boxplot whiskers. Group abbreviations are as defined in Figure 1.

The Shannon index, used to assess microbial diversity and evenness, indicated that rhizosphere soils (BFHR and BFDR) exhibited higher diversity than their corresponding bulk soils (BFHB and BFDB), with BFHR showing the highest value (Figure 2A). Venn diagram analysis revealed that 170 zOTUs (21.1% of the total) were shared across all four soil compartments, representing the core microbial community (Figure 2B). Notably, distinct differences in unique zOTU distributions were observed: diseased rhizosphere soils (BFDR) and healthy rhizosphere soils (BFHR) shared 160 zOTUs, yet harbored 560 and 360 unique OTUs, respectively. In contrast, the bulk soils contributed fewer unique zOTUs, with BFDB and BFHB accounting for 369 and 311 unique zOTUs, respectively.

### 3.3 Bacterial community structure and clustering analysis

Principal coordinate analysis (PCoA) based on Bray–Curtis dissimilarity was performed to assess shifts in soil bacterial community structure between healthy and diseased samples. The analysis revealed significant shifts in bacterial community composition across both bulk and rhizosphere soils (PERMANOVA, *R*^2^ = 0.82, *p* = 0.001; Figure 2C), with PCo1 and PCo2 explaining 42.8 and 27.1% of the total variation, respectively. Permutational analysis of multivariate dispersions (BETADISP, *p* = 0.27) confirmed that these differences were primarily driven by true biological variation rather than differences in within-group dispersion.

PCo1 effectively separated rhizosphere soils (BFHR and BFDR) from bulk soils (BFHB and BFDB), highlighting the dominant influence of soil compartment on microbial community structure. Along PCo2, the two bulk groups clustered closely, suggesting minimal compositional differences, whereas BFHR and BFDR were clearly separated, indicating that root rot markedly alters the rhizosphere bacterial community. These findings were further supported by hierarchical clustering analysis (Figure 2D), where samples grouped first by soil compartment and then by plant health status, with BFDR forming a distinct cluster.

### 3.4 Changes in microbial community composition

**Figure 3:**
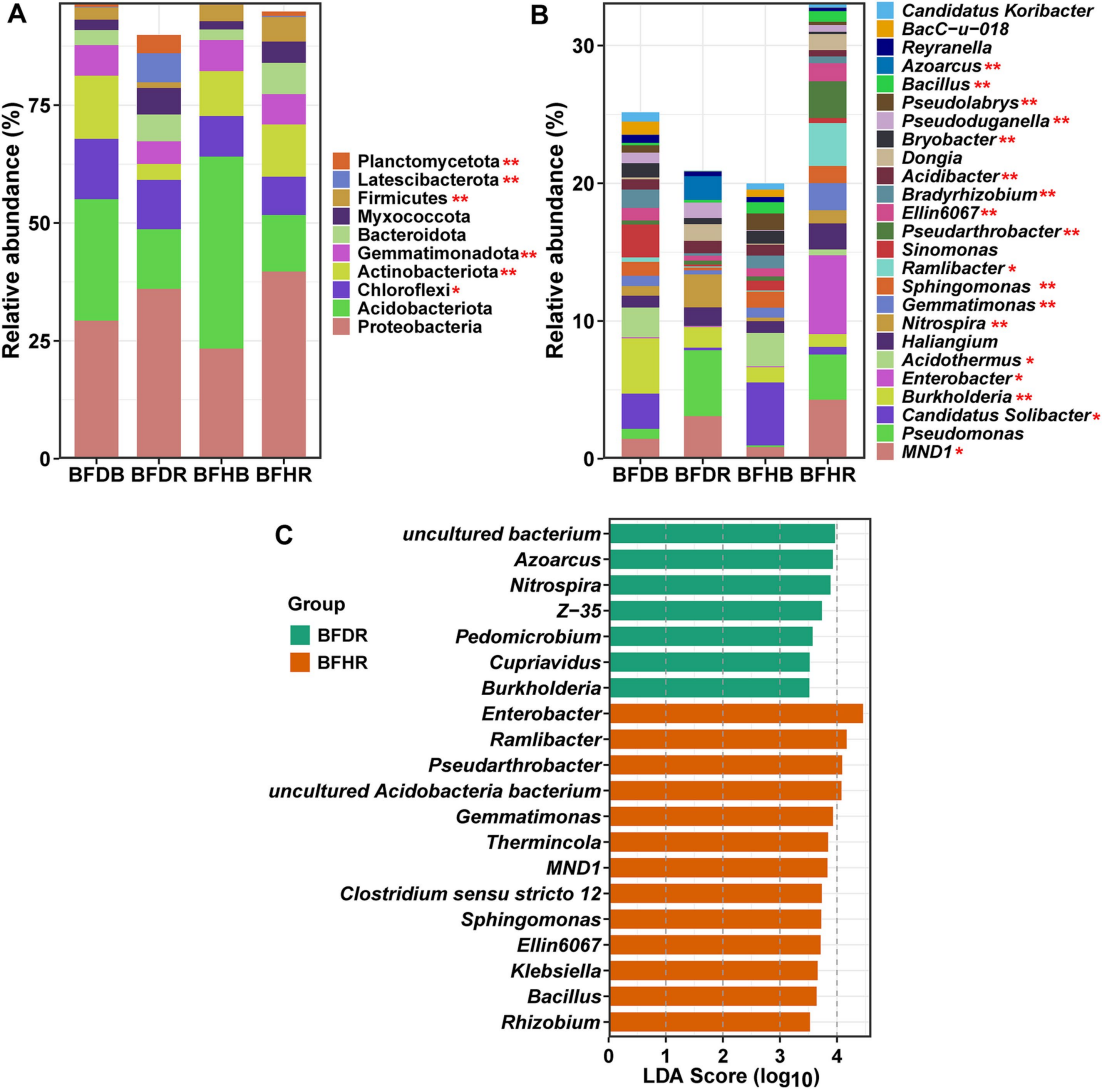
Bacterial community composition and differential taxa analysis. **(A)** Relative abundances of the top 10 bacterial phyla across the four soil treatments. **(B)** Relative abundances of the top 25 bacterial genera. **(C)** LEfSe (LDA effect size) analysis at the genus level, highlighting taxa differentially enriched in the diseased rhizosphere soil (BFDR) versus the healthy rhizosphere soil (BFHR). Asterisks denote significant differences between BFDR and BFHR (*t*-test): “*” denotes *p* < 0.05; “**” denotes *p* < 0.01. Group abbreviations are as defined in Figure 1.

The bacterial community in avocado rhizospheres underwent substantial restructuring under *Fusarium*-induced root rot stress. At the phylum level, both healthy and diseased soils were dominated by Proteobacteria (32.09%), Acidobacteriota (22.77%), Chloroflexi (9.99%), and Actinobacteriota (9.34%) (Figure 3A). Notably, Proteobacteria were more abundant in rhizosphere soils (BFHR and BFDR) compared to bulk soils, whereas Acidobacteriota were more prevalent in the bulk compartments. Diseased rhizosphere soils (BFDR) exhibited significantly higher (*p* < 0.05) abundances of Chloroflexi (10.47 ± 1.57% vs. 8.11 ± 0.68%) and Latescibacterota (6.17 ± 0.39% vs. 0.20 ± 0.11%) than healthy rhizosphere soils (BFHR) (*p* < 0.05). Conversely, Actinobacteriota (11.09 ± 1.72% vs. 3.36 ± 0.69%), Gemmatimonadota (6.45 ± 0.59% vs. 4.83 ± 0.62%), and Firmicutes (5.20 ± 0.54% vs. 1.19 ± 0.48%) were significantly enriched in BFHR (*p* < 0.05).

At the genus level, the 25 most abundant genera are illustrated in Figure 3B. Genera with relative abundances exceeding 1% included *MND1* (2.41%), *Pseudomonas* (2.23%), *Candidatus Solibacter* (1.97%), *Burkholderia* (1.88%), *Enterobacter* (1.48%), *Acidothermus* (1.26%), *Haliangium* (1.24%), and *Nitrospira* (1.08%). Comparisons between diseased (BFDR) and healthy (BFHR) rhizosphere soils revealed that genera such as *Burkholderia*, *Nitrospira*, *Acidibacter*, *Bryobacter*, and *Azoarcus* were significantly more abundant in BFDR. In contrast, *MND1*, *Candidatus Solibacter*, *Enterobacter*, *Acidothermus*, *Gemmatimonas*, *Sphingomonas*, *Ramlibacter*, *Pseudarthrobacter*, *Bradyrhizobium*, *Pseudolabrys*, and *Bacillus* were significantly enriched in BFHR (*p* < 0.05). LEfSe analysis further underscored these distinctions (Figure 3C): in BFHR, genera including *Enterobacter*, *Ramlibacter*, *MND1*, *Sphingomonas*, *Klebsiella*, and *Rhizobium* (within Proteobacteria), *Pseudarthrobacter* (within Actinobacteriota), *Gemmatimonas* (within Gemmatimonadota), and *Bacillus* (within Firmicutes) were significantly enriched, whereas in BFDR, genera such as *Azoarcus*, *Burkholderia*, and *Pedomicrobium* (within Proteobacteria) as well as *Nitrospira* (within Nitrospirota) were more abundant.

### 3.5 Changes in the relative abundance of beneficial bacteria

**Figure 4:**
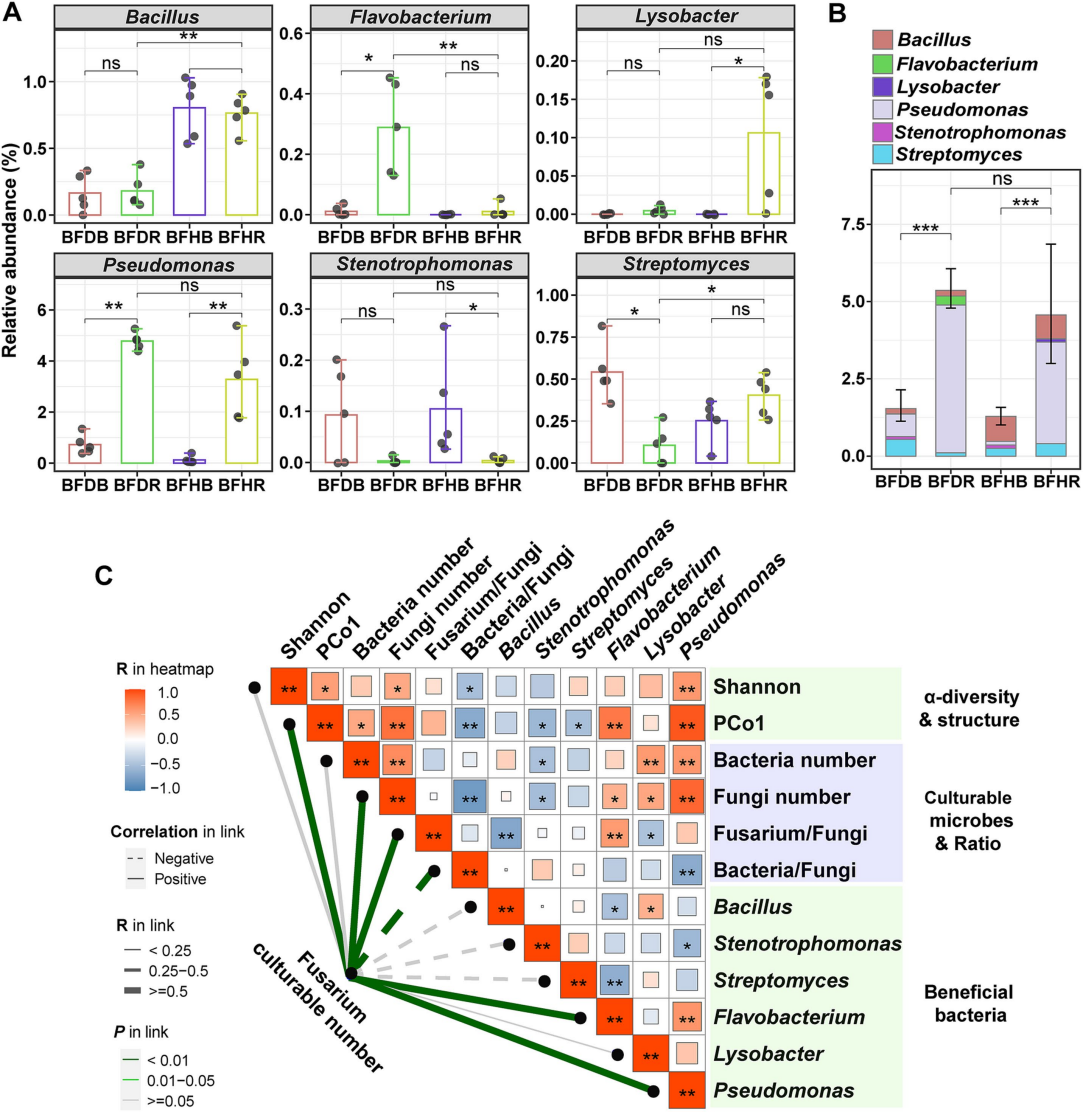
Changes in the relative abundances of beneficial bacteria and correlation analysis. **(A)** Changes in the relative abundances of beneficial bacteria across the four soil treatments. **(B)** Changes in the cumulative abundances of beneficial bacteria. **(C)** Spearman correlation analysis between *Fusarium* abundance and key microbial parameters. Error bars in **(A,B)** represent the standard deviation (SD) based on biological replicates (*n* = 5). The heatmap values (ranging from blue to red) represent correlation coefficients, with asterisks (* and **) indicating significant correlations at *p* < 0.05 and *p* < 0.01, respectively. In the legend, thicker lines correspond to higher correlation coefficients. Dashed lines indicate negative correlations, while solid lines represent positive correlations. Different line colors indicate varying *p* values. Group abbreviations are as defined in Figure 1.

Root rot has a significant impact on the bacterial community, resulting in changes in the relative abundance of specific beneficial bacteria. Among the six beneficial bacterial genera analyzed—*Bacillus*, *Flavobacterium*, *Lysobacter*, *Pseudomonas*, *Stenotrophomonas*, and *Streptomyces*—marked differences in abundance were observed between diseased and healthy rhizosphere soils, as well as between rhizosphere and bulk soils (Figure 4A).

Overall, *Flavobacterium*, *Lysobacter*, and *Pseudomonas* exhibited higher abundances in rhizosphere soils. In the rhizosphere, *Bacillus*, *Lysobacter*, and *Streptomyces* were more abundant in healthy rhizosphere soil (BFHR) compared to diseased rhizosphere soil (BFDR), with *Bacillus* showing a particularly notable increase (4.22-fold; *p* < 0.01). In contrast, *Flavobacterium* and *Pseudomonas* were more abundant in diseased rhizosphere soil (BFDR) than in healthy rhizosphere soil (BFHR). Regarding cumulative relative abundance, both healthy rhizosphere soil (BFHR: 4.57 ± 1.64%) and diseased rhizosphere soil (BFDR: 5.36 ± 0.46%) showed significantly higher abundances of beneficial bacteria (*p* < 0.001) compared to their corresponding bulk soils (Figure 4B). However, no significant differences were observed between BFHR and BFDR in cumulative relative abundance.

### 3.6 Spearman’s correlation between *Fusarium* abundance and key microbial parameters

As shown in Figure 4C, the culturable abundance of *Fusarium* exhibited significant positive correlations (*p* < 0.05) with several microbial indicators, including the bacterial community structure (as represented by PCo1), the culturable abundance of fungi, and the relative abundances of *Flavobacterium* and *Pseudomonas*. In contrast, the culturable abundance of *Fusarium* was significantly negatively correlated with the relative abundances of *Bacillus*, *Stenotrophomonas*, and *Streptomyces*. Additional analyses revealed that the culturable abundance of fungi was significantly positively correlated with that of bacteria, yet negatively correlated with the ratio of bacteria to fungi. Moreover, the relative abundance of *Bacillus* was significantly negatively correlated with the ratio of *Fusarium* to total fungi.

### 3.7 Isolation, characteristics and preliminary identification of *Bacillus* strains with antagonistic activity

**Figure 5:**
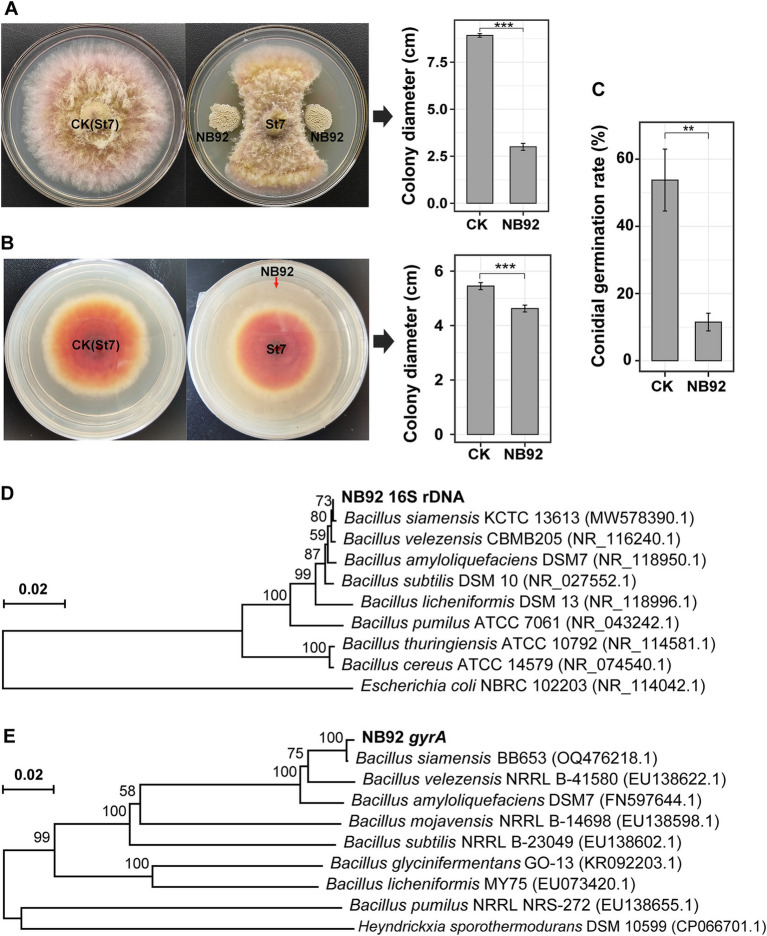
Inhibition of pathogenic fungi (*Fusarium* sp. St7) by NB92 and molecular identification of NB92. **(A)** Inhibition of mycelial growth using a dual culture assay. **(B)** Inhibition of conidial germination by the fermentative liquid produced by NB92. **(C)** Inhibition of mycelial growth of pathogenic fungi by the volatile organic compounds (VOCs) produced by NB92. Error bars in the bar charts and represent the standard deviation (SD) based on biological replicates (*n* = 5). Asterisks indicate statistical significance (*t*-test): “**” denotes *p* < 0.01; “***” denotes *p* < 0.001. **(D,E)** Phylogenetic tree of NB92 based on 16S rRNA and *gyrA* gene sequences.

Given the observed negative correlations between *Fusarium* abundance and the relative abundance of *Bacillus* (and other beneficial genera), along with the higher *Bacillus* levels in healthy avocado soils, we inferred that certain *Bacillus* strains may help suppress root rot pathogens. To test this, 32 *Bacillus*-like isolates with distinct colony morphologies were obtained from healthy rhizosphere soil. Their antagonistic potential against *Fusarium* sp. St7 was assessed using a dual-culture plate assay, identifying six strains with varying levels of inhibitory activity. Phylogenetic analysis of their 16S rRNA gene sequences confirmed their classification as *Bacillus* (GenBank accession numbers: PV465220–PV465225; Supplementary Figure 2). Among these isolates, strain NB92 exhibited the strongest antagonistic activity, achieving a 66.4 ± 2.0% inhibition of the pathogen’s mycelial growth (Figure 5A). Additionally, NB92 produced VOCs that significantly inhibited mycelial growth (Figure 5B) and reduced conidial germination of *Fusarium* sp. St7 (Figure 5C). Based on these promising results, we selected NB92 for further biocontrol studies.

On LB solid medium, NB92 formed milky white, slightly convex colonies (Supplementary Figure 3). Microscopic observation revealed that NB92 comprises motile, rod-shaped, spore-forming cells. Biochemical assays confirmed that NB92 is Gram-positive, tolerates high salt concentrations (growing in LB medium with 5–35‰ NaCl), and can utilize various carbon sources, including glucose, xylose, fructose, sugarcane, and mannitol. Phylogenetic trees based on both 16S rRNA and *gyrA* gene sequences (GenBank accession numbers: PV472352) showed that NB92 is most closely related to *Bacillus siamensis*: it exhibited 100% sequence identity with *B. siamensis* KCTC 13613 based on 16S rRNA data (Figure 5D) and 99% sequence identity with *B. siamensis* BB653 based on *gyrA* data (Figure 5E).

In summary, by integrating morphological, biochemical, and molecular evidence, strain NB92 was preliminarily identified as *B. siamensis*.

### 3.8 Effects of *B. siamensis* NB92 application on *Fusarium* and *Bacillus* counts, and VOC production in soil

**Figure 6:**
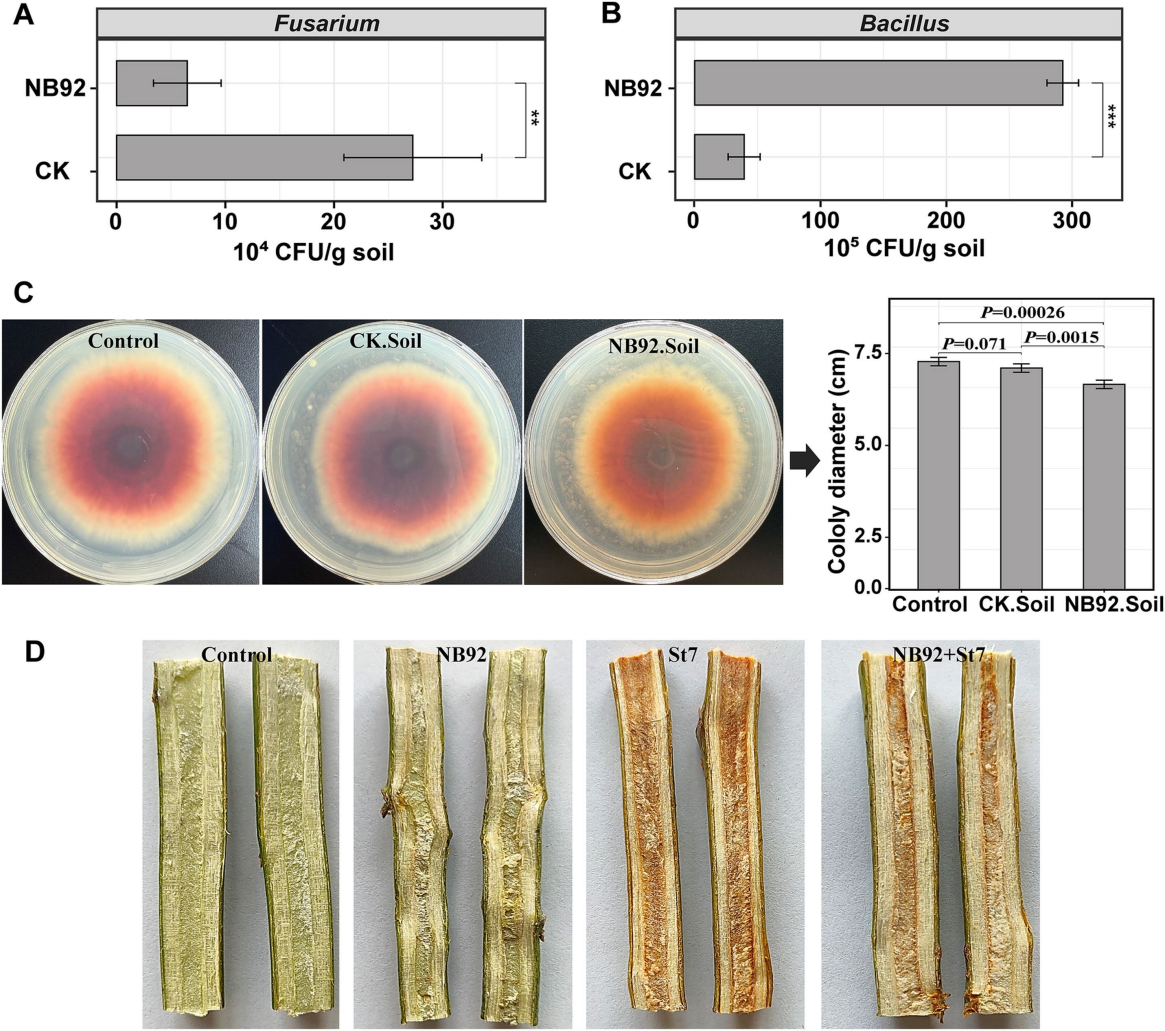
Effects of *B. siamensis* NB92 application on *Fusarium* and *Bacillus* counts, VOC production in soil, and disease protection in avocado. **(A,B)** Changes in *Fusarium* and *Bacillus* counts in diseased rhizosphere soil following NB92 application. **(C)** Inhibition of *Fusarium* sp. St7 mycelial growth by VOCs produced in soil treated with NB92. **(D)** *Ex vivo* stem inoculation assay showing the necrotizing ability of *Fusarium* sp. St7 and the protective effect of NB92 on avocado stem segments. Error bars in the bar charts represent the standard deviation (SD) based on biological replicates (*n* = 5). Abbreviations in panel **(C)**: Control: *Fusarium* only; CK. Soil: soil without NB92; NB92. Soil: NB92-inoculated soil. Abbreviations in panel **(D)**: Control, stem segments treated with sterile water; NB92, stem segments treated with NB92 only; St7, stem segments inoculated with *Fusarium* sp. St7; NB92 + St7, stem segments pretreated with NB92 and subsequently inoculated with *Fusarium* sp. St7.

Following the application of *Bacillus* NB92 to avocado diseased rhizosphere soil (BFDR), the number of cultivable *Fusarium* and *Bacillus* was assessed (Figures 6A,B). *Fusarium* was significantly reduced in the soil after NB92 application [*p* < 0.01; CK: (27.2 ± 6.3) × 10^4^ CFUs/g soil vs. NB92: (6.5 ± 3.1) × 10^4^ CFUs/g soil], while *Bacillus* counts showed a significant increase [*p* < 0.001; CK: (39.2 ± 12.7) × 10^5^ CFUs/g soil vs. NB92: (292.0 ± 12.6) × 10^5^ CFUs/g soil]. Furthermore, NB92 inoculation in the soil led to a significant increase in VOC production (Figure 6C). The VOCs produced in soil with NB92 inoculation significantly (*p* < 0.01) inhibited the mycelial growth of *Fusarium* sp. St7 (NB92. Soil), compared to the pathogen alone (Control) or soil without NB92 application (CK. Soil).

To further evaluate the biocontrol potential of *Bacillus NB92* against avocado root rot, an *ex vivo* stem segment inoculation assay was conducted. As shown in Figure 6D, both the control group (treated with sterile water) and the NB92 group (treated with NB92) exhibited healthy stem tissue. In contrast, the St7 group (treated with the pathogen) displayed significant necrosis, with necrotic lesions extending through the vascular tissue. However, when NB92 was applied prior to pathogen inoculation, the necrotizing ability of *Fusarium* sp. St7 was effectively inhibited, with less damage observed in the stem tissue.

## 4 Discussion

Avocado root rot continues to pose a substantial challenge to sustainable production, a situation aggravated by continuous monoculture and intensive agricultural practices that destabilize the natural soil microbiome equilibrium. Given the relatively stable structure of bacterial communities and their critical contributions to soil ecosystem resilience, this study investigated bacterial community dynamics in avocado soils affected by *Fusarium*-induced root rot, aiming to identify key taxa with biocontrol potential. Our findings revealed that root rot significantly alters the diversity and composition of rhizosphere bacterial communities. These changes underscore the pivotal role of beneficial bacteria in disease suppression and provide a foundation for the development of targeted, microbiome-informed biocontrol strategies for managing avocado root rot.

### 4.1 Shifts in bacterial community structure

Principal coordinate analysis and hierarchical clustering revealed significant shifts in the bacterial community structure between healthy and diseased soils. The rhizosphere communities of diseased soils (BFDR) were markedly different from those of healthy soils (BFHR), with PCo1 effectively distinguishing rhizosphere from bulk soils and PCo2 further differentiating healthy from diseased rhizospheres. This clustering highlights the rhizosphere as a dynamic hotspot for microbial activity and community restructuring during disease progression. In contrast, bulk soil microbes, serving as reservoirs for rhizosphere communities, maintained relative stability (Berendsen et al., 2012).

Analysis of the dominant bacterial phyla revealed that Proteobacteria were more abundant in rhizosphere soils compared to bulk soils, whereas Acidobacteriota predominated in bulk soil compartments. This distribution pattern supports the ecological theory that copiotrophic taxa such as Proteobacteria are favored in nutrient-rich environments like the rhizosphere, while oligotrophic groups such as Acidobacteriota tend to dominate nutrient-poor bulk soils (Pianka, 1970). Furthermore, when comparing the rhizospheres of healthy and diseased plants, the relative abundance of Actinobacteriota and Firmicutes was significantly higher in healthy rhizospheres (BFHR) than in diseased rhizospheres (BFDR). Of particular concern, the diseased rhizosphere showed a marked decline in beneficial genera such as *Bacillus* (Firmicutes) and *Streptomyces* (Actinobacteriota), both known for their biocontrol properties (Compant et al., 2025). These alterations in microbial composition are consistent with patterns observed in other soilborne disease systems (Wang et al., 2025) and are likely a result of pathogen-induced disturbances, including altered root exudation profiles and direct microbial antagonism. These factors collectively suppress beneficial microbial populations and promote the proliferation of opportunistic taxa. Our findings align with those of Solís-García et al. (2020), who similarly reported reductions in Actinobacteria and *Bacillus* spp., accompanied by an enrichment of pathogenic fungi such as *Fusarium* in the rhizosphere of root rot-affected avocado trees.

### 4.2 Alterations in microbial diversity and distribution

Our analysis of alpha diversity using the Shannon index revealed that both healthy and diseased rhizosphere soils exhibited higher diversity than bulk soils, which aligns with the well-documented enrichment effect of plant roots on soil microorganisms (Berendsen et al., 2012). However, when comparing healthy and diseased rhizospheres, the healthy soils (BFHR) showed a significantly higher Shannon index, reflecting a bacterial community with greater richness and evenness. Such a balanced microbial composition is critical for maintaining key soil functions, including nutrient cycling and pathogen suppression (Mallon et al., 2015). In contrast, the diseased rhizosphere (BFDR) exhibited a distinct shift in community structure and reduced diversity, potentially undermining microbial balance and functional stability.

Venn analysis further highlighted that, although a common microbial core exists across soils, the rhizosphere compartments—particularly under diseased conditions—were associated with a greater number of unique zOTUs. This increase may be attributed to the selective recruitment of stress-adapted or opportunistic bacteria, including taxa with potential pathogenic or pathogen-facilitating functions (Li et al., 2021). However, despite the higher number of unique taxa observed in diseased soils, the overall Shannon index decreased, indicating a loss of evenness within the community. This trend suggests that specific genera, such as *MND1*, *Pseudomonas*, and *Nitrospira*, may become disproportionately abundant under disease conditions. Such taxonomic shifts can reduce functional redundancy and destabilize microbial networks, thereby diminishing the resilience of the rhizosphere microbiome. Ultimately, this imbalance may impair critical ecosystem functions, including nutrient cycling and pathogen suppression, thus exacerbating the negative impacts of root rot on avocado health (Huang et al., 2019; Wang et al., 2024a).

### 4.3 Role of beneficial bacteria in disease suppression

Our correlation analyses revealed a significant negative correlation between the bacteria-to-fungi ratio and culturable *Fusarium* abundance, emphasizing the importance of maintaining a balanced microbial community in the rhizosphere. A higher bacteria-to-fungi ratio in healthy soils suggests the presence of a robust bacterial community associated with lower pathogen levels. Notably, the relatively stable structure of the bacterial community provides a solid foundation for effective biocontrol strategies, and the overall abundance of culturable bacteria is critical for disease suppression (Bonanomi et al., 2010). Previous studies have also shown that fungi colonizing plant roots often possess pathogenic potential, whereas bacterial communities are essential for sustaining host health (Duran et al., 2018).

In our study, the relative abundance of beneficial genera, such as *Bacillus* and *Streptomyces*, was higher in healthy rhizosphere soils compared to diseased ones. Similarly, other studies have shown that soils enriched with antagonistic bacteria like *Bacillus*, *Streptomyces*, and *Pseudomonas* often exhibit reduced disease incidence (Compant et al., 2025). Additional beneficial biomarkers in healthy rhizosphere soil, such as *Rhizobium* and *MND1*, play a crucial role in the soil nitrogen cycle by promoting nitrogen fixation, transformation, and enhancing nitrogen uptake by plants, thereby improving soil fertility (Fang et al., 2024). These results support the hypothesis that beneficial bacteria are essential for maintaining a balanced microbial community capable of suppressing pathogenic fungi, likely through mechanisms such as competition for nutrients and space, antibiosis, and the induction of plant defense responses (Chen et al., 2019). For example, *Bacillus subtilis* strains isolated from the avocado rhizoplane have demonstrated effective biocontrol activity against soil-borne pathogens such as *F. oxysporum* and *Rosellinia necatrix* (Cazorla et al., 2007).

Interestingly, our data also indicate that although certain beneficial bacteria (e.g., *Flavobacterium* and *Pseudomonas*) are enriched in diseased soils, the overall reduction in key beneficial groups such as *Bacillus* in the rhizosphere of diseased plants points to a potential “cry for help” mechanism (Santhanam et al., 2015; Arnault et al., 2023). In this scenario, stressed plants may attempt to modify their rhizosphere microbiome to recruit beneficial microbes and counteract pathogen invasion. However, the lower abundance and reduced competitive ability of these recruited beneficial bacteria compared to dominant pathogens might limit their disease control efficacy. Future research should investigate whether the exogenous application of beneficial bacterial inoculants can enhance this natural recruitment process and reinforce the soil’s defense capabilities.

### 4.4 Characterization and antagonistic potential of *B. siamensis* NB92 against *Fusarium*

Guided by our correlation analyses and the higher relative abundance of *Bacillus* in healthy rhizospheres, as well as its well-established antagonistic properties (Zhang et al., 2023), we isolated several *Bacillus* strains for further evaluation. Among these isolates, strain NB92 exhibited the highest inhibition rate against the root rot pathogen (*Fusarium* sp. St7). Morphological, biochemical, and molecular analyses, including 16S rRNA and *gyrA* gene sequencing, identified NB92 as closely related to *B. siamensis*. *Bacillus* strains are renowned for producing antifungal lipopeptides such as iturin, fengycin, and surfactin that inhibit pathogenic fungi (Zhang et al., 2023).

In recent years, *B. siamensis* has garnered attention as an effective biocontrol agent against *Fusarium*-induced plant diseases across diverse cropping systems. For instance, Zhang et al. (2024) reported that *B. siamensis* strain QN2MO-1, isolated from tomato rhizosphere, produced chitinase and β-1,3-glucanase, effectively reducing postharvest and planting-stage tomato *Fusarium* wilt. Similarly, Hussain et al. (2024) demonstrated that wheat rhizosphere isolate *B. siamensis* Sh420 synthesized potent antifungal lipopeptides that inhibited *F. graminearum*. In chickpea, Gorai et al. (2023) found that endophytic *B. siamensis* CNE6 suppressed *Fusarium solani* through production of inhibitory metabolites and activation of plant defense genes. In banana, *B. siamensis* strain Gxun-6 was shown to exhibit broad-spectrum antifungal activity, including up to 88% control efficacy against *Fusarium oxysporum* (Shen et al., 2022). Likewise, Sharma et al. (2021) isolated endophytic *B. siamensis* NKIT9 from tomato seeds, which exhibited strong lipopeptide biosynthesis and antagonism against several fungal pathogens. These studies, combined with our findings, reinforce the broad antifungal potential of *B. siamensis* strains and highlight NB92 as a novel and promising addition to this growing list—particularly for use in microbiome-informed strategies for managing avocado root rot.

In addition to inhibiting *Fusarium* growth and conidial germination via diffusible compounds, VOCs produced by *Bacillus* strains—demonstrated here in both pure culture and soil inoculation experiments—also play a significant role in pathogen suppression. These VOCs can diffuse through the soil matrix and modulate microbial interactions over considerable distances, offering an effective, non-contact strategy for biocontrol. This finding is consistent with previous reports showing that VOCs emitted by rhizobacteria, including those from avocado roots, can inhibit *Fusarium* spp. and other soil-borne pathogens (Méndez-Bravo et al., 2018). Although we did not chemically characterize the VOCs emitted by strain NB92, our two-sealed plate assay confirmed their inhibitory effect on *Fusarium* sp. St7, indicating that NB92 volatiles are bioactive under *in situ* conditions. Previous studies have identified several antifungal VOCs produced by *Bacillus* spp. (including *B. siamensis*), such as 2,3-butanedione (diacetyl), acetoin, various ketones, benzenoids, alcohols, pyrazines, and sulfur-containing compounds, which are known to suppress fungal pathogens at low concentrations (Wang C. et al., 2022; Wang D. et al., 2022; Grahovac et al., 2023). These volatiles suppress fungal pathogens through multiple mechanisms, including disruption of membrane integrity, inhibition of metabolic activity, attenuation of virulence traits, and priming of host immune responses. Given the precedent set by these known compounds and the strong inhibition observed in our assays, it is plausible that NB92 produces a similar VOC profile. Future studies involving GC–MS or other metabolomic approaches will be essential to identify the active volatiles and facilitate the development of VOC-based biocontrol formulations.

Furthermore, the observed increase in *Bacillus* counts, accompanied by a decrease in *Fusarium* abundance following the application of strain NB92, suggests that this bacterium not only directly antagonizes pathogens but also effectively colonizes the soil and potentially stimulates indigenous beneficial bacterial populations. This enhanced microbial balance may contribute to a more resilient and disease-suppressive soil environment. Moreover, the application of NB92 effectively inhibited the necrotizing ability of *Fusarium* sp. St7. Collectively, these findings underscore the potential of *Bacillus* NB92 as a promising biocontrol agent for avocado root rot, functioning through both direct pathogen inhibition and enhancement of a beneficial soil microbiome.

### 4.5 Implications for soil health and disease management

The restructuring of the bacterial community observed in our study emphasizes the intricate interactions within the avocado rhizosphere. In healthy rhizosphere soils, a balanced microbial community—characterized by higher diversity, a favorable bacteria-to-fungi ratio, and the presence of beneficial taxa such as *Bacillus*—plays a critical role in preventing pathogen establishment and controlling disease progression. This balance is essential for maintaining soil health, supporting nutrient cycling, and enhancing disease resistance. In contrast, root rot disease disrupts this equilibrium, resulting in an overrepresentation of pathogenic fungi and a depletion of beneficial microbes, which exacerbates disease severity. Our findings underscore the importance of fostering a healthy and diverse microbial community as a key strategy for effective disease management in avocado orchards.

One promising biocontrol strategy emerging from our study involves the use of antagonistic *Bacillus* strains, such as *Bacillus* NB92. However, it is essential to recognize that using a single strain in controlled experiments may not fully replicate the complexities and variability encountered in natural field conditions. Microbial communities in the field are influenced by a range of environmental factors, and microbial interactions are multifaceted. Therefore, a more sustainable and effective approach might involve the use of microbial consortia—combinations of multiple beneficial bacterial strains. Such an approach can harness the synergistic effects of different strains, leading to more robust and long-term disease suppression. This strategy, which advocates for multi-strain inoculants to create a “disease-suppressive soil” (Santhanam et al., 2015), can enhance the resilience of the soil microbiome and improve its capacity to suppress pathogenic microorganisms.

While our study provides compelling evidence for the role of *Bacillus* spp. in suppressing *Fusarium* populations in avocado rhizosphere soils, several important limitations must be addressed. Firstly, field trials are essential to validate the effectiveness of *Bacillus* NB92 in controlling avocado root rot under real-world agricultural conditions. Such trials would provide crucial insight into the practical applicability and reliability of *Bacillus* NB92 as a biocontrol agent for root rot management. Additionally, although our correlation analyses suggest that beneficial bacteria, particularly *Bacillus* spp., are linked to lower *Fusarium* abundance, the underlying mechanisms remain unclear. The production of antimicrobial compounds, including diffusible metabolites and VOCs, is likely a significant factor in pathogen suppression. However, further research is needed to identify these compounds and elucidate their modes of action. Advanced techniques such as metabolomic profiling and gene expression analysis will be essential for understanding how *Bacillus* NB92 and similar strains interact with root pathogens at the molecular level. This deeper understanding will be crucial for developing more targeted, effective, and sustainable biocontrol strategies.

## 5 Conclusion

Our study demonstrates that avocado root rot significantly disrupts the rhizosphere bacterial community structure, resulting in reduced microbial diversity and a marked decline in key beneficial taxa, such as *Bacillus* and *Streptomyces*. The observed negative correlations between *Fusarium* abundance, the bacteria-to-fungi ratio, and the relative abundance of beneficial bacteria highlight the critical role these microbes play in pathogen suppression. Building on these findings, we isolated *Bacillus* strain NB92, which exhibited strong antagonistic activity against the root rot pathogen through both direct inhibition and VOC production. Application of NB92 to diseased rhizosphere soil led to increased *Bacillus* counts and a reduction in *Fusarium* abundance. Furthermore, NB92 was effective in inhibiting the necrotizing ability of the pathogen. These results underscore the potential of leveraging beneficial bacteria as a sustainable biocontrol strategy for managing avocado root rot. Our findings provide valuable insights into microbiome-based disease management, offering a basis for future research on biocontrol. Subsequent studies, including the development of multi-strain inoculants, field trials, and mechanistic investigations (e.g., identification of active diffusible metabolites and VOCs), will be crucial for advancing effective biocontrol strategies in avocado orchards.

## Data Availability

The datasets presented in this study can be found in online repositories. The names of the repository/repositories and accession number(s) can be found at: https://www.ncbi.nlm.nih.gov/, PRJNA1247462.
