# Supplementary material for: Bacterial community shifts in Fusarium-induced avocado root rot and the antagonistic potential of Bacillus siamensis NB92
Source: Front Microbiol. 2025 Jun 18;16:1626537. doi: 10.3389/fmicb.2025.1626537 (PMC12213771; doi:10.3389/fmicb.2025.1626537)
Supplement: Supplementary file 1 [file Table_1.docx]

Supplementary Material


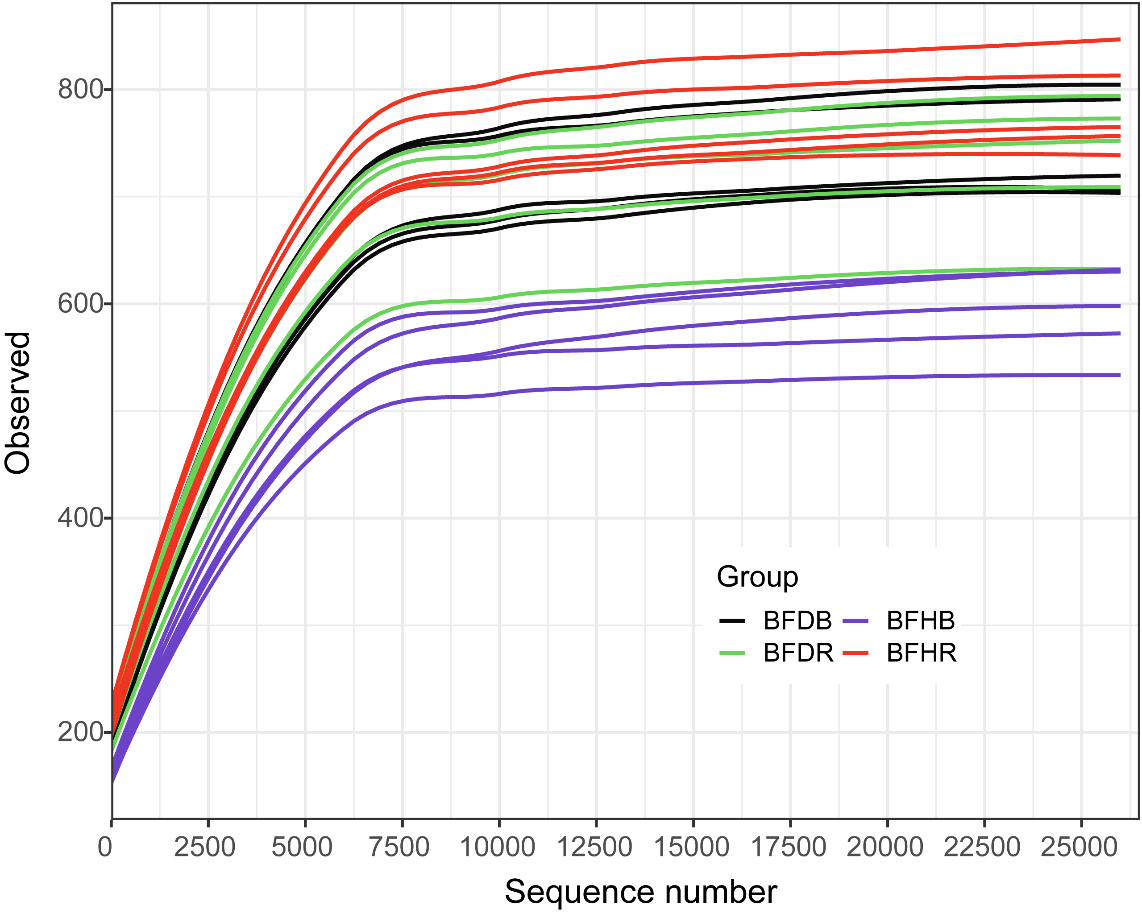


**Supplementary Figure 1.** Rarefaction curve for each sample based on 16S rRNA sequencing.


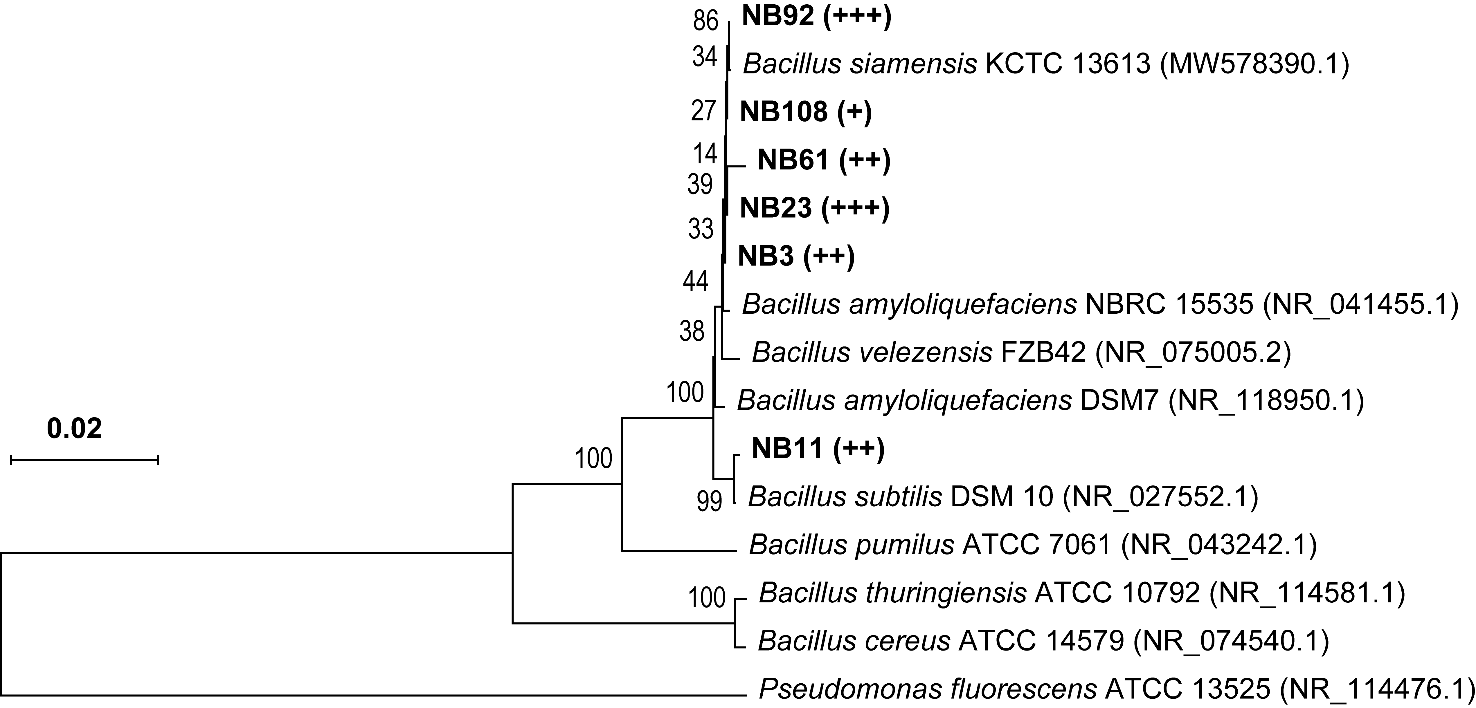


**Supplementary Figure 2.** Phylogenetic analysis of 16S rRNA gene in the six candidate *Bacillus* strains and their antagonistic inhibition rates against the root rot pathogens (*Fusarium* sp. St7). The inhibition rates of the six *Bacillus* strains against *Fusarium* sp. St7 were indicated alongside each strain name and categorized as follows: “+” for 20–40%, “++” for 40–60%, and “+++” for greater than 60%.


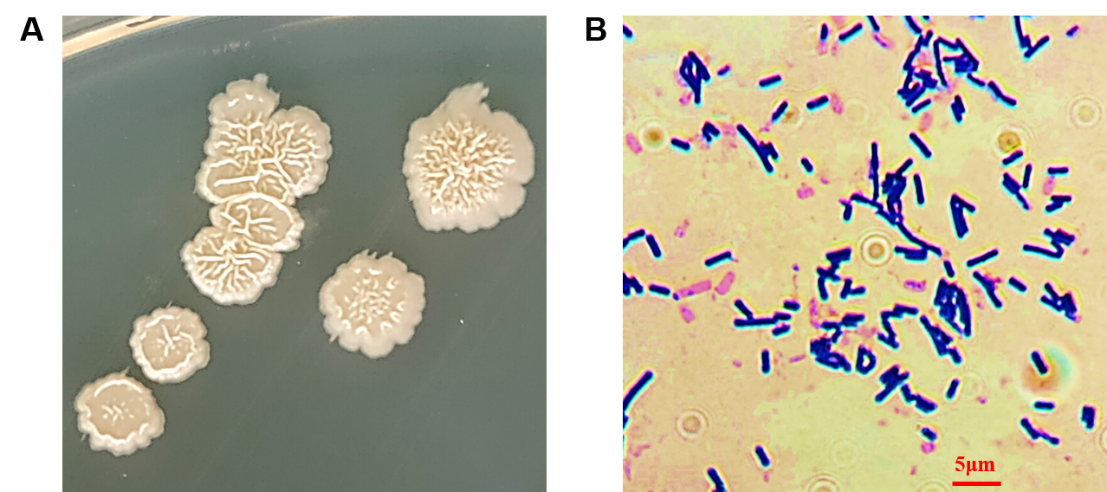


**Supplementary Figure 3.** Single colony morphology (A) and Gram-stained microscopic morphology (B) of strain NB92.
